# Additive Routes to Action Learning: Layering Experience Shapes Engagement of the Action Observation Network

**DOI:** 10.1093/cercor/bhv167

**Published:** 2015-07-24

**Authors:** Louise P. Kirsch, Emily S. Cross

**Affiliations:** 1 Wales Institute for Cognitive Neuroscience, School of Psychology, Bangor University, Bangor, UK; 2 Department of Social and Cultural Psychology, Behavioural Science Institute, Donders Institute for Brain, Cognition and Behaviour, Radboud University Nijmegen, Nijmegen, The Netherlands

**Keywords:** action observation network, dance, motor learning, parietal, premotor, training

## Abstract

The way in which we perceive others in action is biased by one's prior experience with an observed action. For example, we can have auditory, visual, or motor experience with actions we observe others perform. How action experience via 1, 2, or all 3 of these modalities shapes action perception remains unclear. Here, we combine pre- and post-training functional magnetic resonance imaging measures with a dance training manipulation to address how building experience (from auditory to audiovisual to audiovisual plus motor) with a complex action shapes subsequent action perception. Results indicate that layering experience across these 3 modalities activates a number of sensorimotor cortical regions associated with the action observation network (AON) in such a way that the more modalities through which one experiences an action, the greater the response is within these AON regions during action perception. Moreover, a correlation between left premotor activity and participants' scores for reproducing an action suggests that the better an observer can perform an observed action, the stronger the neural response is. The findings suggest that the number of modalities through which an observer experiences an action impacts AON activity additively, and that premotor cortical activity might serve as an index of embodiment during action observation.

## Introduction

When watching another person perform an action, whether and how we have previously experienced that action has the potential to profoundly shape how that action is perceived. For example, when watching a flamenco dancer perform, it is possible for an observer to be an aficionado of flamenco guitar music, or an avid spectator at flamenco performances, or perhaps even a flamenco dancer herself. As this example illustrates, action experience can be acquired through a number of different avenues, including physical practice, visual experience, or exposure to the music that accompanies action. Prior work demonstrates that an action observation network (AON), comprising sensorimotor brain regions including premotor, parietal, and occipitotemporal cortices that are engaged when watching others in action, responds more robustly when observing actions that have been physically practised (Calvo-Merino et al. 2005; Cross et al. 2006; Lee and Noppeney 2011; Liew et al. 2013; Rüther et al. 2014; Woods et al. 2014) or visually experienced (Mattar and Gribble 2005; Torriero et al. 2007; Cross et al. 2009; Jastorff et al. 2009; Jola et al. 2012), compared with similar actions with which participants have no prior experience.

In the auditory domain, listening to the sounds associated with specific actions also results in greater AON engagement compared with listening to sounds that accompany unfamiliar, unrehearsed actions. This has been demonstrated in music (Lahav et al. 2007; Callan et al. 2010) and sporting domains (Woods et al. 2014), as well as with single-neuron recordings within the premotor cortex (PMC) of nonhuman primates (Keysers et al. 2003; Ferrari et al. 2005). Together, these findings suggest that the AON operates in a supramodal manner, such that the modality through which a previously learned action is perceived is less important than whether or not an action has been performed in the past. While this research provides evidence for sensorimotor engagement when listening to sounds that are a direct result of performing a particular action, the influence of sounds that do not directly result from action execution on AON engagement remains unexplored (such as the soundtrack that might accompany particular movements, like guitar music accompanying flamenco dancing). Given the less direct link between action and audition in such scenarios, we might expect that experience with sounds that could accompany (but are not the direct result of) action might also engage the AON.

One way of conceptualizing how different sensory cues could lead to activation of sensorimotor brain regions is based on Hebbian learning theory (Hebb 1949; Keysers and Gazzola 2014). According to this theory, synapses become stronger “when one cell repeatedly assists in firing another” (Hebb 1949, p. 63). As such, the existence of multisensory/multimodal mirror neurons (Keysers et al. 2003) could, in part, be explained by correlated firing patterns of auditory and motor or visual and motor neurons during action performance and action perception. We can speculate that populations of neurons composing parts of the AON possess similar properties, and that learning reinforces neural connections, leading to more detailed action representations and improved execution abilities (see Keysers and Gazzola 2014). The aim of the present study is to explore how action representations are ‘built up’ from one modality to several. Based on prior work comparing the impact of visual experience only with visual plus physical experience (e.g., Calvo-Merino et al. 2006; Aglioti et al. 2008; Cross et al. 2009; Makris and Urgesi 2015), and Hebbian learning theory (Hebb 1949; Keysers and Gazzola 2014), we predict that by increasing the modalities through which a participant experiences a novel action, concomitant increases in AON engagement during action observation and physical performance ability should occur.

In the present study, a group of dance-naïve participants learned a series of complex, whole-body movement sequences via an interactive dance video game. We probed how action representations change as types of experience are combined, as assessed by brain and behavioral measures. We focus on action learning in 3 modalities: auditory, visual, and motor. Our main question concerns how experience built up across these 3 modalities shapes de novo action representations for learners of a fast-paced dance video game. The simplest, unimodal condition features only auditory information (the “A condition”), where participants spent time each day listening to the soundtrack that accompanied a dance music video, with no concurrent visual or motor experience with the dance movements. To this, we added a layer of action information with visual cues of the specific movements associated with the music (the visual/auditory or “VA condition”). Finally, the most complex condition combined physical, visual and auditory experience (the “PVA condition”). For this condition, participants trained to perform dance sequences set to music videos, and their performance ability was assessed with basic motion capture technology. Importantly, participants spent an identical amount of time experiencing sequences from all 3 training conditions. A separate set of dance music videos was left entirely untrained to more sensitively probe the impact of experience within each of these modalities. Immediately prior to and following 4 days of training, participants underwent identical functional magnetic resonance imaging (fMRI) scanning sessions. Such a design enabled investigation of the impact of unimodal and multimodal training conditions on action observation, as well as how an individual's ability to perform a complex action relates to brain activity.

We aimed to address 2 specific questions concerning how experience within the auditory, visual, and motor domains shapes newly learned action representations at brain and behavioral levels. First, we ask how increasing the number of modalities through which an action is learned shapes brain activity, and whether any regions associated with the AON are sensitive to the layering or addition of the types of training experience we experimentally manipulate. We predict AON activity will increase as the number of learning modalities increases in the following manner: untrained ≤ A < VA < PVA. While it is likely that different modalities have different effects on AON activity and learning, we specifically selected these 4 conditions in order to demonstrate the greatest linear spread between different kinds of training experience. If this hypothesis were supported, it would be in line with previous behavioral results (Kirsch et al. 2013), as well as prior work that has compared physical and visual experience with certain actions to similar untrained actions (Calvo-Merino et al. 2006; Cross, Cohen et al. 2012; Jola et al. 2012; Liew et al. 2013). Next, we explore the extent to which learning from physical and audiovisual experience together differs to learning from audiovisual experience only. While some literature suggests that both kinds of experience can impact parietal and premotor regions of the AON in a similar manner (Frey and Gerry 2006; Cross et al. 2009), direct comparison of both conditions should illuminate what is special about physical practice, per se. Based on prior work (Cross et al. 2009), we expect more premotor activation when observing PVA compared with VA sequences. With our approach, we can also evaluate the extent to which physical performance of the trained sequences correlates with the magnitude of neural signal during action observation. In keeping with previous studies (Beilock et al. 2008; Cross et al. 2009; Liew et al. 2013), we predict a positive relationship between objective physical ability and neural activity to emerge within premotor and parietal cortices.

## Materials and Methods

### Participants

Twenty-two physically and neurologically healthy young adults were recruited from the Bangor University student population. All participants were reimbursed for their involvement with either cash or course credit and provided written informed consent before taking part. The Bangor University School of Psychology research ethics committee approved all components of this study. Only dance-naïve participants were selected. This meant all participants had limited or no experience performing or observing dance, and none had prior experience playing dance video games. All participants were right-handed. Two participants were excluded from the final sample due to excessive motion artifacts while undergoing fMRI scanning. The final sample of 20 participants comprised 12 females with a mean age of 23.4 years (standard deviation = 4.1 years).

### Stimuli and Apparatus

Eight dance sequences from the dance game “Dance Central 2” (Harmonix Music Systems 2011) for the XBox 360 Kinect™ console were chosen that featured gender-neutral dance movements. The 8 chosen dance sequences were specifically selected so as to contain no overlapping dance moves between songs (i.e., each move was uniquely associated to one song/dance sequence). Each dance sequence was set to a popular song (e.g., *Like a G6* by Far East Movement or *Hot Stuff* by Donna Summer) and varied in length from 2:05 to 2:35 min, with a mean length of 2:19 min. The accompanying music varied in tempo from 95 to 130 beats per minute (BPM; mean = 115.8 BPM). To focus participants' attention on the avatar whose moves they were learning, the same background setting was selected for all dance videos, which had a minimal amount of extraneous movement. The difficulty of the dance sequences (complexity and amplitude of dance movements) was set to a medium level to ensure participants could perform them but would be challenged across the training period. The 8 dance sequences were paired to create 4 groups whose composition was matched for number and complexity of specific dance movements, as well as BPM. Each pair of sequences was assigned to one of the four training conditions: physical, visual, and auditory experience (PVA), visual and auditory experience (VA), auditory experience only (A), and no experience/untrained (UNT). A total of 4 different training groups were assembled, meaning that each pair of dance sequences was trained in all 4 training conditions across participants.

For the fMRI portion of the experiment, 64 short dance segments with accompanying soundtracks were extracted from the 8 full dance sequences using iMovie ‘11 (Apple, Inc.), 8 from each full dance sequence. The resultant 64 stimuli were between 3.5 and 4.5 s in length (mean = 3.95 s) with half the stimuli featuring the female avatar and half featuring the male avatar. Each stimulus was edited so that it featured one complete, coherent dance move involving whole-body motion and significant spatial displacement of the limbs (cf. Calvo-Merino et al. 2008). To obtain a task-specific visual baseline, 10 extra stimuli of the avatar standing in place (5 s) were created by capturing video footage from the end of the sequence when the avatar had finished dancing and stood still, but features in the background were still moving. All stimuli were novel to the participants during the pretraining fMRI scan.

#### Behavioral Training Procedure and Analysis

**Figure 1.**
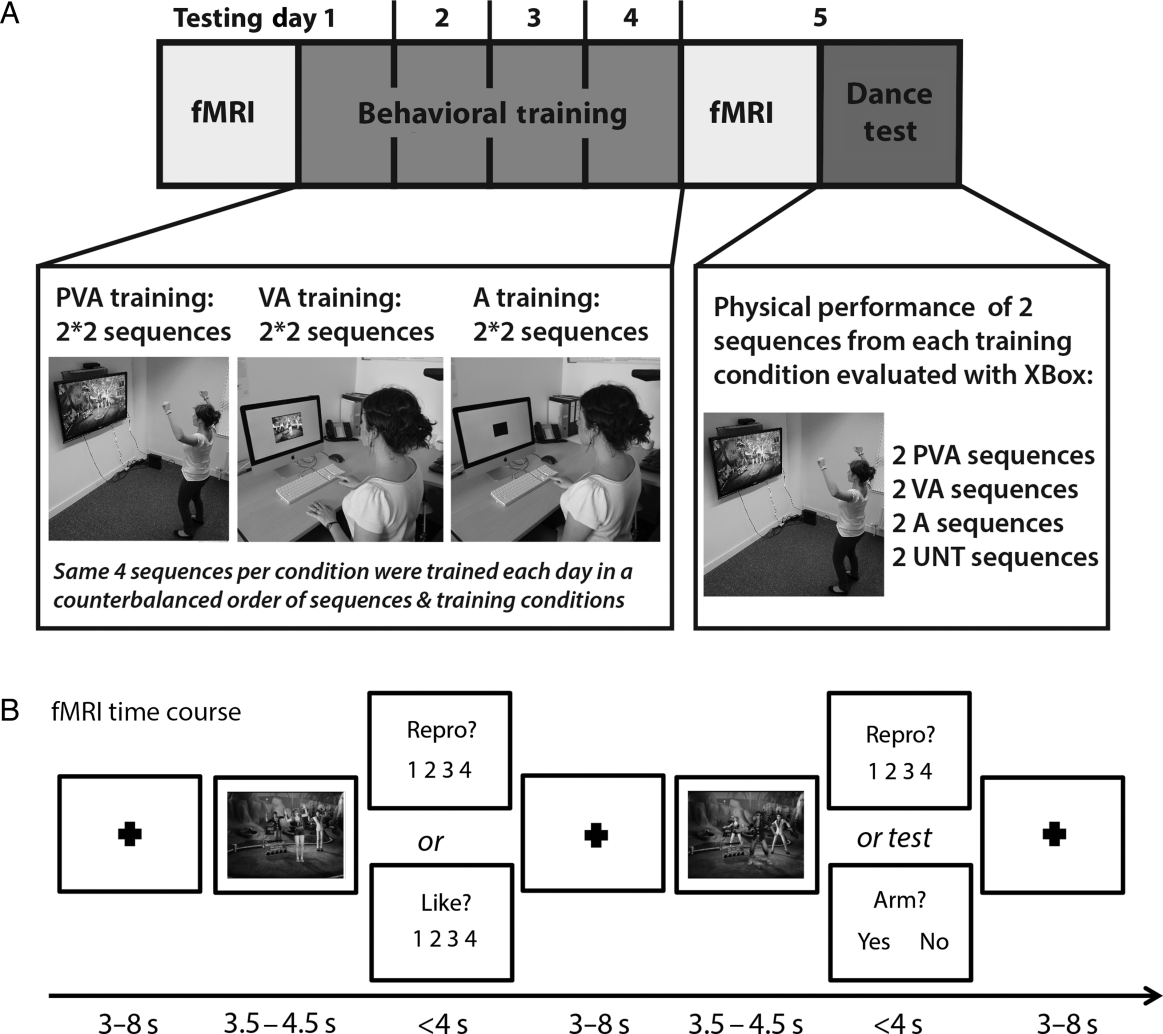
(*A*) Experimental design depicting the phases of the study in chronological order. All participants completed 2 identical fMRI sessions, 4 consecutive days of behavioral training and a final dance test. Representation of the 3 training conditions: physical, visual, and auditory experience (PVA), visual and auditory experience (VA), auditory experience only (*A*). Participants learned 2 distinct sequences in each training condition, but practiced or watched/listened to each sequence twice on each of the 4 days of training (for the PVA and VA conditions, once with a male and once with a female avatar). For the dance test on Day 5, participants performed all 8 sequences once in a counterbalanced order. (*B*) Time course of each fMRI trial. After a fixation screen (length pseudo-logarithmically randomized between 3 and 8 s), participants watched a 5-s dance movement, and were then asked to rate each movement on one of two dimensions: either “How much did you like the movement you just watched?” or “How well could you reproduce the movement you just watched.” Participants responded via a button press, on 4-point Likert scale. The question remained on the screen until a response was made or a maximum of 4 s. Six additional videos randomly appeared and were followed by one of several attentional control questions that required a yes or no response (e.g., “Did the dancer place at least one arm above their head?”).

Participants were randomly assigned to one of the four training groups in which they experienced the same pairs of sequences assigned to the 3 training conditions (PVA, VA, and A) across 4 consecutive days of training. The 4 days of training took place between the pre- and post-training fMRI scanning sessions (Fig. 1). For each training session, participants completed PVA, VA, and A training on the set of sequences to which they had been randomly assigned. Participants physically practiced their 2 PVA sequences twice (once with a female and once with a male avatar), observed and listened to 2 VA sequences twice, and listened twice to the soundtracks from the 2 A training sequences (with no visual input). The order in which participants completed the training conditions was counterbalanced within and between participants across training days. Each training session lasted approximately 40 min.

##### Physical + visual + auditory experience

For sequences participants physically practiced, they stood approximately 2 m away from a 52” Sharp flat screen television mounted on the wall in front of them. Participants' task was to mirror the dance movements of the avatar in the “Dance Central 2” Xbox 360 game and concentrate on improving their performance during subsequent sessions. The Kinect™ motion capture system compared participants' movements with the avatar's movements and assigned a score based on accuracy of mirroring the avatar. The Kinect™'s scoring system is based on synchrony, speed, and kinematics of the participant's movements, but as it is a closed system consumer product, further details about how scores are assigned were not available. However, such specifications are not critical for the present study, as we are mainly interested in relative performance differences before and after training. Participants received on-screen feedback about how well they performed in the form of a final score after each sequence. Participants' dance scores were recorded by the researcher and used as the objective measure of dance performance for the behavioral analyses.

The 4 raw scores participants received each day for the dance sequences in the PVA condition were averaged so that each participant had a single score representing dance performance for each training day. A repeated-measures ANOVA with training day as a within-subjects factor with 4 levels (Days 1, 2, 3, and 4) was conducted on these scores to confirm the training manipulation worked and that physical performance increased across the daily training sessions. Additionally, we performed pairwise comparisons, correcting for multiple comparisons, to determine how performance on consecutive days of training compared.

##### Visual + auditory experience

For the sequences for which participants acquired visual and auditory experience, they sat comfortably in front of a computer running Psychophysics Toolbox 3 in MATLAB R2010a (Mathworks, Inc.), which presented the full dance videos. Each video was shown twice, once for each avatar, in a random order. The dimensions of the dance videos were 640 × 480 mm, which reflected perceptually similar scaling to the physical training condition. Participants listened to the soundtrack that accompanied each sequence via the computer speakers. Participants were instructed to pay close attention to the dance sequences, and were told that they would have to perform the sequences at the end of the week, so they should try to learn the movements. To test that they were paying close attention, at the end of each music video, 10 short dance segments (5 from the videos they had just watched) were displayed, without music, each followed by the question “Did you see this movement in the video you just watched?” Participants had to respond “yes” or “no” using the keyboard arrow keys. All test videos were presented silently (as the task would have been too easy if the accompanying soundtracks were also presented).

An accuracy score for each participant on each of the 4 days of training was calculated based on their performance on this task, and a repeated-measures ANOVA on these accuracy scores was conducted to investigate the effect of VA training on the recognition task accuracy over the days of training. Additionally, we performed pairwise comparisons, correcting for multiple comparisons, to determine how performance on the VA task compared across consecutive days of training.

##### Auditory experience only

For the sequences that participants received only auditory experience with, they sat at a computer running MATLAB R2010a and Psychophysics Toolbox 3, which presented the 2 dance video soundtracks twice each in a random order. Visually, participants saw only a black screen and were instructed to listen carefully to the music. To ensure participants paid attention to the music, a short beep was randomly interspersed within the music (10 beeps per sequence) to which the participants had to respond, using the right arrow of the keyboard. On average, participants responded accurately to 99% of the beeps, with no differences between individual songs or across training days.

### Post-training Performance Assessment

On the final day of the study (Day 5), after all other experimental procedures were completed, participants returned to the laboratory to perform the 6 full sequences used in training (PVA, VA, A trained sequences), as well as the 2 untrained sequences (segments of which they had observed during both fMRI sessions). The test followed the same paradigm as the PVA training phase of the study: participants physically performed the dance sequences from all 8 songs, mirroring the avatar's dance movements, while the Kinect™ system captured and scored their movements. The 8 sequences were randomized and balanced for the gender of the avatar. Objective performance scores were obtained in the same way as for the PVA training condition.

Raw scores from both exemplars from each training category were averaged within training conditions to produce an average score per participant for each of the four test conditions. We performed a repeated-measures ANOVA on these scores to investigate the impact of different kinds of experience on physical performance. Pairwise comparisons (Bonferroni corrected for multiple comparisons) were subsequently evaluated to further investigate any differences between conditions in more detail. Degrees of freedom reflect the Greenhouse–Geisser correction, where sphericity has been violated.

#### Neuroimaging Procedure

Each participant completed one fMRI session prior to the training procedures and an identical session immediately following the 4 days of training. Participants completed 2 runs within each scanning session, lasting an average of 15 min and containing 80 trials each. In each run, participants watched and listened to 64 music video stimuli featuring short dance segments taken from the 4 training conditions (PVA, VA, A, and untrained) that were each between 3.5 and 4.5 s in length. Each stimulus was preceded by a fixation cross presented for 3–8 s (the amount of time the fixation cross was on the screen was pseudo-randomized). Each trial was followed by one of two questions in which participants were required to aesthetically rate the observed dance movement (How much did you “like” the movement you just watched?), or assess their physical ability to reproduce the movement (How well could you “reproduce” the movement you just watched?). These questions were shortened to “LIKE?” and “REPRODUCE?,” respectively, and participants responded via a button response. The next trial started once participants answered or after a maximum of 4 s. Participants provided their response via a four-button fiber optic response box placed on their lap on which they rested the index finger and middle fingers of both hands over the buttons. The Likert scale ranged from 1 (not at all) to 4 (extremely), and was counterbalanced across participants such that the scale was reversed for half of the participants. Participants were instructed to watch the dance movements carefully and respond to the question following each video. Analyses that take into account participants' ratings were the focus of a separate study (Kirsch et al. 2015). Ten additional video stimuli featuring the main dancer standing still were presented throughout the functional runs for 5 s each and required no response. Finally, 6 additional video stimuli (that were not part of the full set of 64 videos from the training conditions) were included for attentional control questions. After each of these six test trials, participants were asked a question that required a yes (Button 1) or no (Button 4) response (reverse order counterbalanced between participants). This question was, “Did the dancer place at least one arm above their head?,” and was designed to ensure the participants paid full attention to the dancer's movement in each stimulus. Participants were familiarized outside the scanner prior to the pretraining scan with the all features of the experiment and what they would be asked to do while in the scanner.

Stimuli presentation and response recording was done with a Mac desktop computer running MATLAB R2010a (Mathworks, Natick, MA, USA) and Psychophysics Toolbox 3 (Brainard 1997; Pelli 1997; Kleiner et al. 2007). Stimuli were retroprojected onto a translucent screen viewed via a mirror mounted on the head coil. The experiment was carried out in a 3-T Philips MRI scanner using a SENSE phased-array 32-channel head coil. For functional imaging, a single-shot echo planar imaging sequence was used (*T*_2_*-weighted, gradient echo sequence; echo time TE = 30 ms; flip angle, 90°). The scanning parameters were as follows: repetition time TR = 2000 ms; 30 axial slices; voxel dimensions, 3 × 3 mm with voxel slice thickness = 4 mm; slice gap = 0.8 mm; field of view, 230 × 230 × 143 mm; matrix size, 128 × 128 mm^2^; anterior–posterior phase-encoding. Parameters for *T*_1_-weighted anatomical scans were: 240 × 240 mm^2^ matrix; voxel dimensions, 2 × 2 × 2 mm; TR = 12 ms; TE = 3.5 ms; flip angle = 8°. Due to an error in the scanning protocol, for the first 14 scan sessions, brain slices were acquired in an interleaved manner, while the last 26 scan sessions were collected in an ascending order. Any discrepancies between the 2 orders of acquisition were corrected during preprocessing with appropriate slice time correction procedures. For each run of each scanning session, the first 2 brain volumes were discarded to reduce saturation effects. Depending on participants' response time to each question and the pseudorandom duration of the fixation cross prior to each trial, the total number of functional scans collected for each participant ranged between 369 and 480 volumes (mean = 395 volumes) per functional run.

#### fMRI Data Analysis

Neuroimaging data from each scanning session were first analyzed separately. Data were realigned and unwarped in SPM8 (Wellcome Department of Imaging Neuroscience, London, UK) and normalized to the Montreal Neurological Institute (MNI) template with a resolution of 3 × 3 × 3 mm. Slice timing correction was performed after realignment. Functional data were normalized to individual participants' *T*_1_ anatomical scans with a resolution of 3 mm^3^. All images were then spatially smoothed (8 mm). A design matrix was fitted for each participant, with each type of dance video (PVA, VA, A, and UNT conditions), as well as button presses, attentional control videos, still body videos modeled together as a boxcar function convolved with the standard hemodynamic response function.

Random-effects neuroimaging analyses at the group level were designed to achieve 2 main objectives:

##### Parametric effects of layering experience modality

The first analysis evaluated the hypothesis that an increasing number of modalities encountered during training should lead to greater engagement of AON regions. To test this hypothesis in a particular order (from Untrained to A to VA to PVA), we conducted a parametric analysis assigning equal increasing weight as the number of training modalities increases (i.e., UNT = −3; A = −1; VA = 1; PVA = 3). To more fully ensure findings were specifically due to our training manipulation, this analysis was performed as a training experience by scanning session interaction. Specifically, we compared brain activity from pre- and post-training scans in the same analysis by searching for brain regions that demonstrated a greater difference between the 4 training conditions during the post-training scan than during the pretraining scan. Pre- and post-training data were modeled separately at the first level, and then contrasted directly at the group level. As such, this contrast was evaluated as a paired *t*-test contrasting brain regions emerging from the post-training parametric analysis (UNT = −3; A = −1; VA = 1; PVA = 3) > pretraining parametric analysis (UNT = −3; A = −1; VA = 1; PVA = 3). To further explore the nature of the BOLD response within predicted brain regions, parameter estimates were extracted from a sphere with a 3-mm radius centered on the peak voxel from each training condition (PVA, VA, A, UNT), from each scanning session (pre- and post-training scans). We include plots of these parameter estimates created with rfxplot (Gläscher 2009) for illustration purposes only, to clarify how brain responses to the individual training conditions compare within and between scanning sessions.

##### Neural processes common and distinct to PVA and VA training conditions

The next set of analyses focused on the 2 training conditions most commonly examined when investigating the impact of experience on action perception; the PVA and VA conditions. By exploring what is common to both training conditions, we aim to advance understanding of how watching and/or performing a novel, complex action shapes brain activity. To achieve this, we used custom-written MATLAB code to perform a conjunction analysis to reveal brain regions responding to both kinds of training in a similar manner. To perform this conjunction analysis, we looked for areas of overlap between contrasts evaluating a training experience × scan session interaction (see Supplementary Table 2). These contrasts revealed brain regions showing a greater difference when observing trained compared with untrained sequences during the post-training scan compared with the pretraining scan (here again, this is done to control for any spurious differences between these conditions during the pretraining scan). More precisely, this conjunction analysis evaluated overlap between 2 contrasts evaluated as paired *t*-tests comparing the post-training > pretraining scan sessions (Post-training [PVA > UNT] > pretraining [PVA > UNT] and post-training [VA > UNT] > post-training [VA > UNT]). To further explore how brain regions representing PVA and VA experience in a common manner relate to behavior, we next extracted parameter estimates from a sphere with a 3-mm radius centered on the peak voxel from each common region identified by the conjunction analysis, for each training condition. We then conducted correlational analyses between the parameter estimates from each of these two training conditions (PVA and VA) and participants' actual physical performance associated with each kind of training, measured on Day 5. To test the specificity of the training manipulation, we also correlated these parameter estimates with participants' PVA dance scores on Day 1 (the very first time participants attempted to perform these sequences).

Finally, to explore what is unique to physical experience with a complex, full-body action, we evaluated the PVA > VA contrast during the post-training scan. This contrast enables us to explore what physical experience or movement embodiment contributes to action observation per se, in that all other features of the stimuli (including the time spent training with PVA and VA stimuli, and the visual/auditory nature of the stimuli themselves) are held constant.

All neuroimaging analyses were evaluated at the whole-brain level with a voxel-wise threshold of *P* < 0.001 uncorrected and *k* = 10 voxels. We focus on brain regions that reached cluster-corrected significance at the FWE-cluster-corrected *P* < 0.05 level. Anatomical localization of all activations was assigned based on consultation of the Anatomy Toolbox in SPM (Eickhoff et al. 2005, 2006), in combination with the SumsDB online search tool (http://sumsdb.wustl.edu/sums/).

## Results

### Behavioral Results

**Figure 2.**
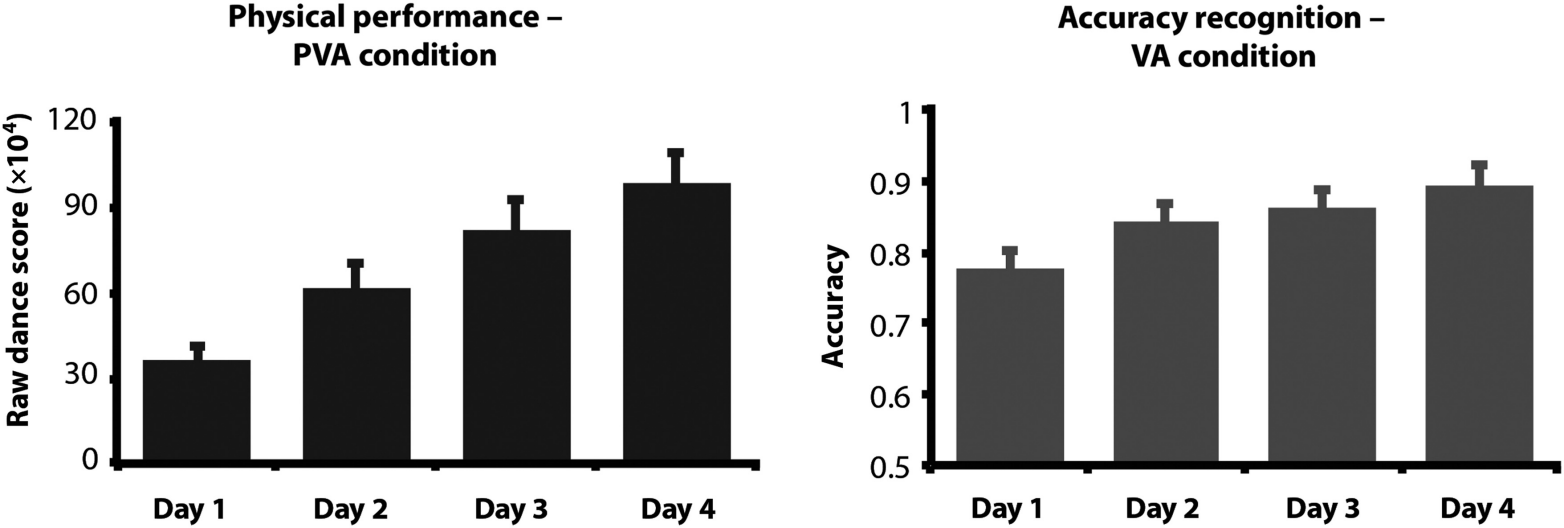
The left plot illustrates mean dance scores for the PVA trained sequences across training days. The right plot illustrates mean accuracy performance on the recognition task for the VA trained sequences. Error bars in both plots indicate the across-subject standard error of the mean.

Participants' physical performance was assessed via 2 sequences performed twice each day across 4 consecutive days of training. A repeated-measures ANOVA revealed significant improvement over the 4 days of training, *F*_1.640,31.159_ = 68.868, *P* < 0.001 (Fig. 2*A*). A linear trend best captured the relationship between training day and performance score, as each day participants' dance scores significantly increased compared with the previous day (all *P* < 0.001).

Participants' attention to the VA training procedures was assessed via a recognition task after watching each full sequence. A repeated-measures ANOVA revealed significant improvement on this movement recognition task over the 4 days of training, *F*_3,57_ = 10.730, *P* < 0.001 (Fig. 2*B*). This demonstrates that participants became better at recognizing the constituent movements from the VA training condition, and consequently appeared to become increasingly (visually) familiar with the VA sequences. Pairwise comparisons demonstrate that the differences between Day 1 and subsequent days' performances differed significantly (*P* = 0.023; *P* = 0.011; *P* = 0.001, respectively), whereas performance differences between Days 2, 3, and 4 did not reach significance (all *P* > 0.07).

**Figure 3.**
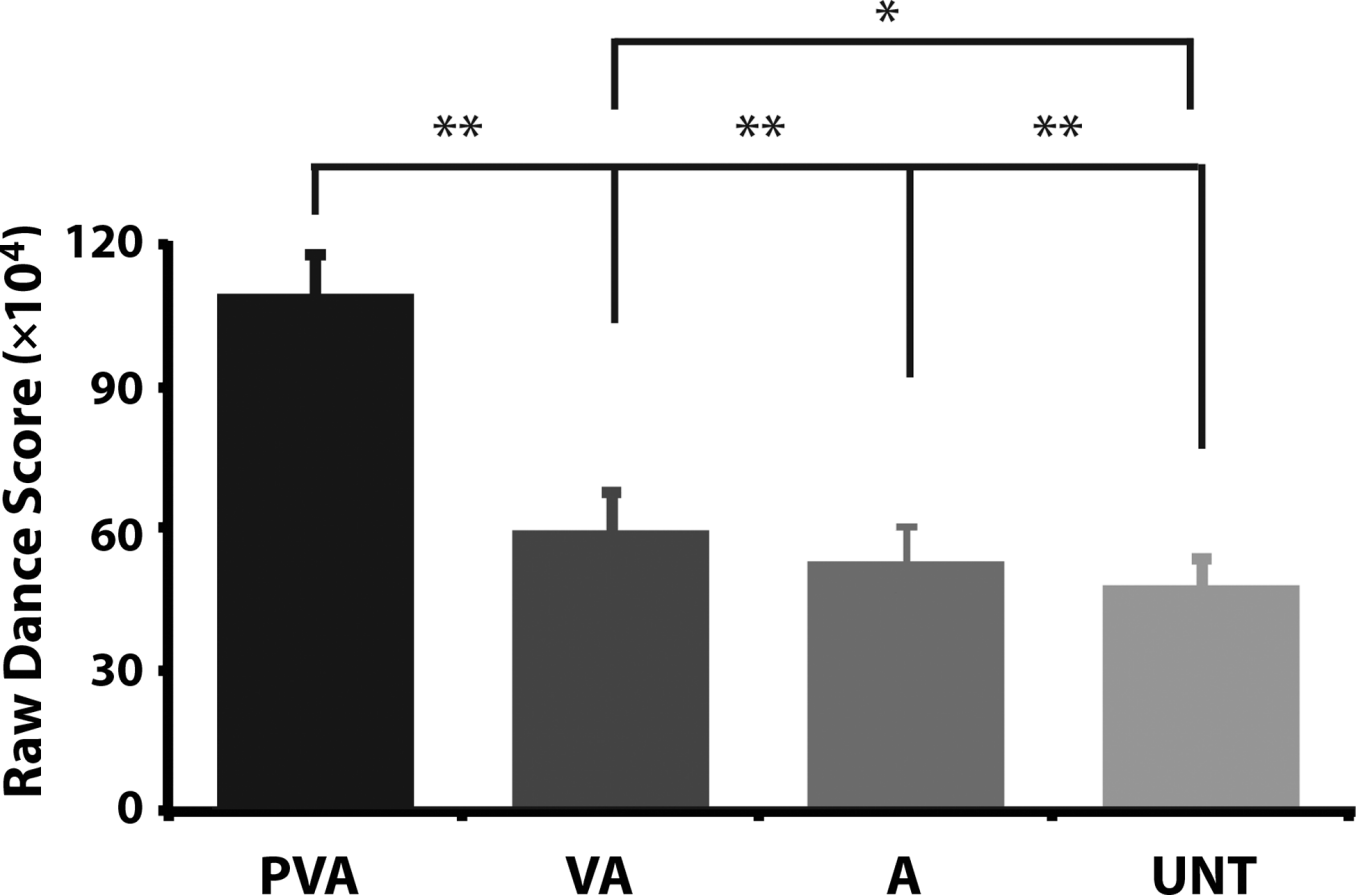
Mean dance scores for all the sequences performed on Day 5, for each training condition. Significant difference between PVA and the other condition was found, as well as difference between VA and UNT conditions. **P < 0.001 and *P < 0.05.

On the fifth and final day of testing, after completing all fMRI procedures, participants returned to the laboratory and physically performed all 8 sequences used in the experiment. They performed each sequence once. Analysis of dance performance scores from this final dance test revealed a significant effect of training condition on dance score, *F*_3,57__=_ 77.861, *P* < 0.001, whereby participants performed sequences they physically practiced throughout the week (PVA condition) significantly better than sequences from every other training condition (all *P* < 0.001), and performed the VA sequences significantly better than untrained sequences *(P* = 0.037). No other differences between individual training conditions were significant. These results are illustrated in Figure 3.

### Functional MRI Results

#### Parametric Effects of Layering Experience Modality

One of our core research questions concerns the extent to which sensorimotor cortical regions respond to the layering of training modalities for a novel action sequence. We hypothesized that not only will some regions within the AON be engaged when observing actions that have been physically practiced, but also that parts of this network should be modulated by the layering of experience types such that having 3 kinds of experience (PVA) will be associated with more activation than 2 kinds (VA), which will show more activation than 1 kind (A), which will, in turn, show more activation than when observing entirely UNT sequences.

**Figure 4.**
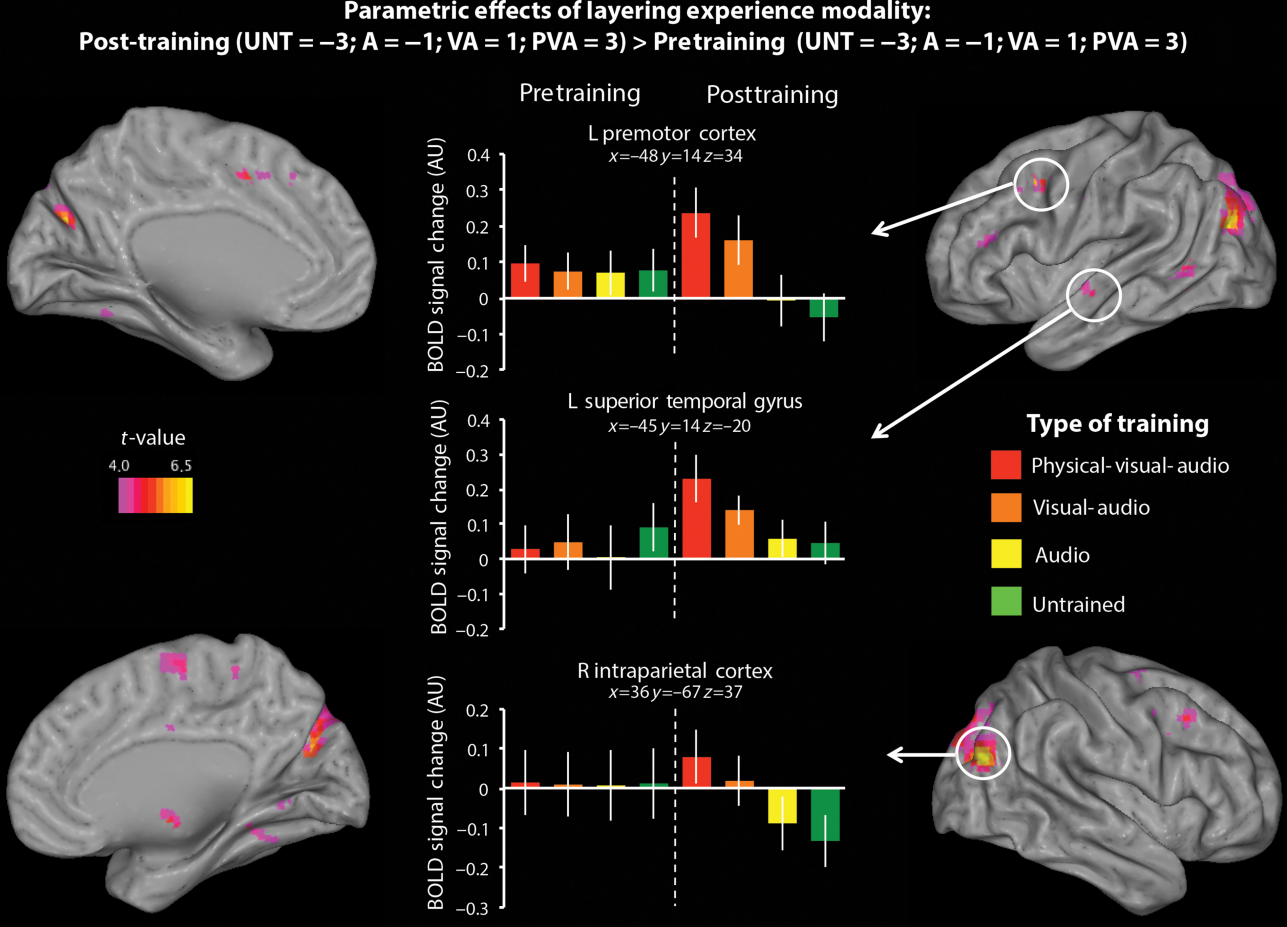
Regions activated with increasing experience with an observed movement. Plots represent the percent signal change (AU: arbitrary units) within 3 AON regions that emerged from this contrast that met the established cluster-corrected threshold (*P*_FWE-corrected_ < 0.05, *k* = 10 voxels; see Table 1 for complete listing of cluster-corrected regions to emerge from this contrast) for each type of stimulus (PVA, VA, A, and UNT), from the pre- and post-training scans. For the left premotor cortex, left superior temporal gyrus, and right intraparietal cortex, *x*, *y*, and *z* values are presented in MNI coordinates. Plots are included for illustration purposes only. Error bars indicate the across-subject standard error of the mean.

Behaviorally, we saw a subtle additive effect of training modality on physical performance (see Fig. 3). This behavioral finding (which replicates that reported by Kirsch et al. 2013) was followed up with a parametric analysis assessing brain regions that showed an interaction between number of training modalities by scanning session (post-training parametric analysis [UNT = −3; A = −1; VA = 1; PVA = 3] > pretraining parametric analysis [UNT = −3; A = −1; VA = 1; PVA = 3]). AON regions that emerged from this contrast included left PMC, left superior temporal gyrus, and right intraparietal cortex (IPC). Figure 4 illustrates these 3 brain regions. The corresponding parameter estimates (included for illustration purposes only) show that each of these brain regions demonstrates a pattern of increasing BOLD activity when watching movements from the UNT to A to VA to PVA conditions in the post-training scan session only. Importantly, and as the plotted parameter estimates in Figure 4 underscore, none of these brain regions discriminated between the training conditions in the pretraining scan session (as would be expected). The same increase in neural response amplitude in relation to layering of experience was also observed in several brain regions outside the AON, including the anterior and posterior cingulate, as well as in the anterior fusiform gyrus (see cluster-corrected regions in Table 1, and full listing of results in Supplementary Table 1).

**Table 1:** Regions associated with an increase of experience and modalities involve in the training, across scan session (Day 5 > Day 1)

Region	BA	MNI coordinates			Putative functional name	*t*-Value	Cluster size	*P*_corr._ value
		*x*	*y*	*z*				
L anterior cingulate gyrus	24/32	−15	32	28		7.01	296	<0.001
R superior frontal gyrus	8/9	21	29	31	SFG	5.66		
R superior frontal gyrus	9	15	41	28	SFG	5.34		
L cingulate cortex/calcarine	18	−12	−58	10		6.85	931	<0.001
R lingual gyrus	17/18	9	−55	7		6.84		
Precuneus	31	0	−67	28	SPL	5.93		
L middle occipital gyrus	39	−45	−73	25	IPC	6.22	413	<0.001
L inferior parietal lobule	7	−39	−55	46	IPL	4.42		
L angular gyrus	39	−57	−58	25	IPC	4.33		
R middle occipital gyrus	39	36	−67	37	IPC	6.07	130	0.005
R middle occipital gyrus	19	39	−70	28	IPC	4.82		
R middle temporal gyrus	39	54	−64	22	MTG	4.72		
L fusiform gyrus	20	−24	−34	−20		6.00	74	0.042
L fusiform gyrus	20	−30	−25	−26		4.67		
L fusiform gyrus	20	−36	−43	−26		3.60		
L inferior frontal gyrus	44	−48	14	34	PMC	5.82	649	<0.001
L inferior frontal gyrus	45	−51	23	16	IFG	5.60		
L inferior frontal gyrus	44	−51	8	22	IFG	5.56		
L temporal pole	38	−45	14	−20	STG	5.71	110	0.010
L inferior frontal gyrus	47	−27	11	−23		4.75		
L medial temporal pole	38	−39	14	−29		4.23		
L posterior cingulate cortex	23	−9	−34	28		4.88	188	<0.001
R posterior cingulate cortex	23	6	−16	28		4.65		
R posterior cingulate cortex	23	6	−28	28		4.46		

BA, Brodmann area; R, right; L, left; IFG, inferior frontal gyrus; IPC, intraparietal cortex; IPL, inferior parietal lobule; MTG, middle temporal gyrus; PMC, premotor cortex; SFG, superior frontal gyrus; SPL, superior parietal lobule; STG, superior temporal gyrus.

Significance at all sites for each contrast was tested by a one-sample *t*-test on beta values averaged over each voxel in the cluster, *P* < 0.001, uncorrected; *k* = 10 voxels. Up to 3 local maxima are listed when a cluster has multiple peaks more than 8 mm apart. Only regions that reached a FWE-cluster-corrected threshold of *P* < 0.05 are reported in this table.

#### Neural Processes Common and Distinct to PVA and VA Training Conditions

**Figure 5.**
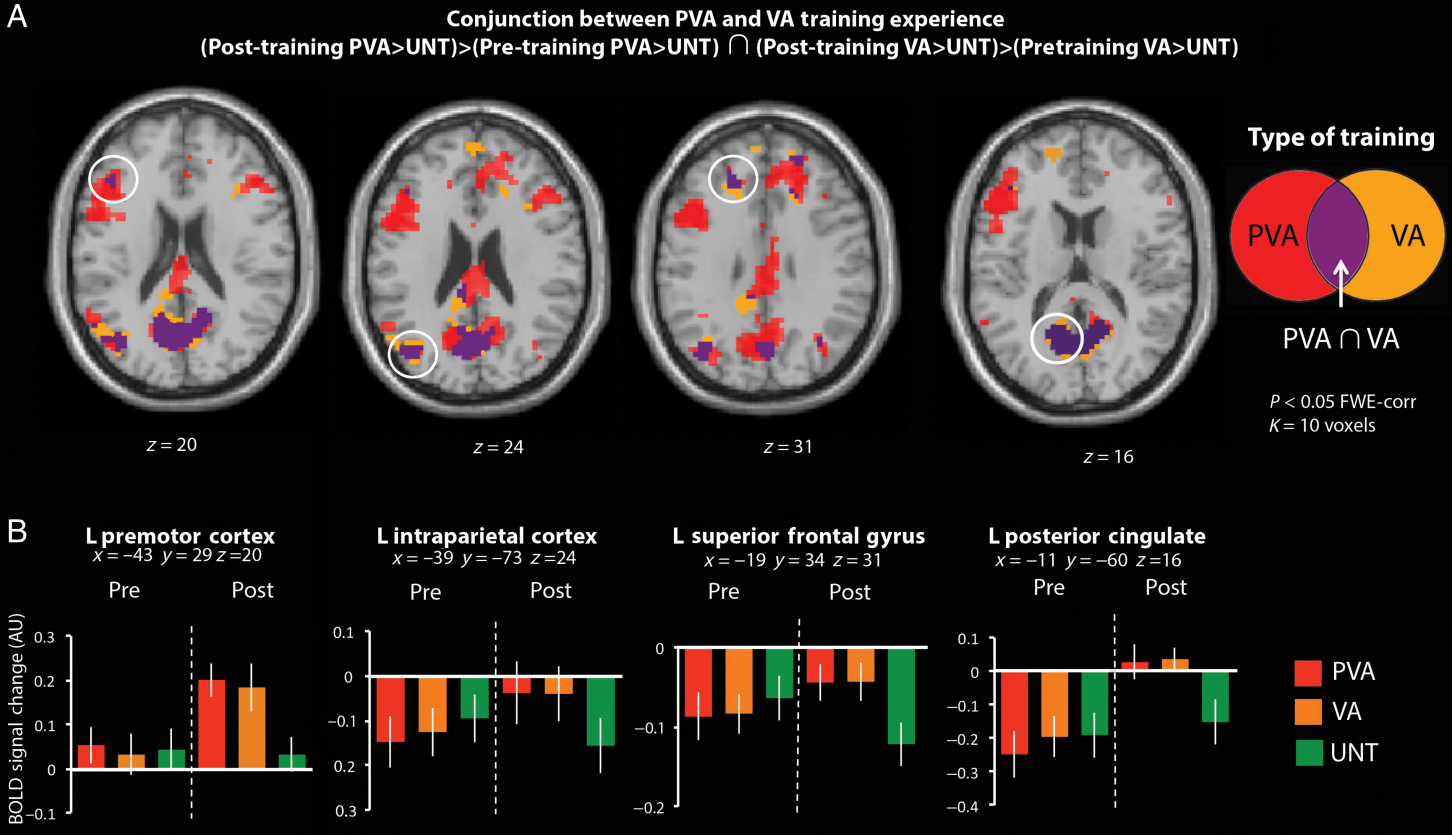
Conjunction analysis illustrating brain regions common to the PVA > UNT and VA > UNT contrasts, across scan session. (*A*) Four regions showed overlap with PVA and VA experience, including left premotor cortex, left intraparietal cortex (IPC), left superior frontal gyrus (SFG), and posterior cingulate cortex. (*B*) Plots show the percent BOLD signal change (in arbitrary units) for each condition in these 4 common regions that met the established cluster-corrected threshold (*P*_FWE-corrected_ < 0.05, *k* = 10 voxels), for each scanning session and are included for illustration purposes only. Error bars indicate the across-subject standard error of the mean.

In order to further explore the contribution of PVA and VA experience on AON activity (in relation to the untrained condition), we next conducted a conjunction analysis (Fig. 5). This analysis revealed regions of neural overlap between the scan session by training experience interactions for the PVA > UNT and VA > UNT contrasts. As such, this approach illustrates how visual and visuomotor experience with a complex movement sequence compare.

This analysis revealed 4 brain regions spanning 495 voxels common to PVA and VA experience, including PMC, IPC, superior frontal gyrus (SFG), and posterior cingulate cortex, all in the left hemisphere (Fig. 5*A*). To more clearly visualize the response of these 4 regions when watching actions from the different training conditions, the parameter estimates from each of the 3 conditions (PVA, VA, and UNT) were extracted from the pre- and post-training scan sessions and plotted in Figure 5*B*. The plots illustrate that each of these brain regions shows a response of similar amplitude to PVA and VA experience, and a decreased response when viewing untrained stimuli, only after the training intervention.

**Figure 6.**
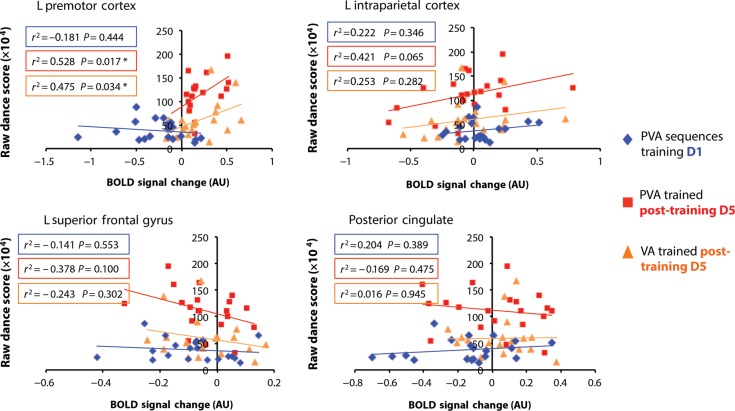
Correlation between percent BOLD signal change (in arbitrary units) and participants' physical scores on Day 1 for PVA sequences to be trained and on Day 5 for PVA and VA conditions for the 4 common regions to PVA and VA training (left premotor cortex, left intraparietal cortex, left superior frontal gyrus, and posterior cingulate). Parameter estimates were extracted from a 3-mm sphere centered on the peak of each region. *P < 0.05.

To explore the relationship between neural activity common to PVA and VA training and participant's actual physical performance, we next correlated participants' dance scores from the PVA and VA trained sequences on Day 5, as well as their scores on the PVA sequences from Day 1 (when all sequences were new and participants performed them for the first time). Results show that participants' first attempt at performing these sequences (performance on Day 1) did not correlate with the magnitude of neural responses when they watched these same sequences for the first time in the scanner (Fig. 6; data points plotted with blue diamonds). However, “after” 1 week of training, the magnitude of activity in left PMC significantly correlated with participants' performance scores, but does not seem to discriminate between the PVA and VA training conditions. Of note, IPS showed a similar shift toward a positive correlation between BOLD signal change and raw dance score for PVA sequences only, although this correlation did not reach significance (*P* = 0.065). Neither left SFG nor the posterior cingulate showed any significant relationship to participants' physical performance post-training (Fig. 6). This pattern of findings suggests that the response within left PMC might relate to participants' overall ability to physically reproduce a movement acquired after 4 days of training (whether in the physical or observational domain), but future work with larger sample sizes will be required to replicate this result and validate this hypothesis.

The final set of analyses directly assessed brain activity when participants observed dance segments from the PVA compared with VA training conditions during the post-training scan session. This analysis enabled us to ask which, if any, brain regions are specially tuned to represent physical experience (or the addition of physical experience to visual and auditory experience). While no brain region survived the stringent statistical threshold of *P* < 0.05 FWE-corrected, one brain region did emerge at *P* = 0.088, FWE-corrected. This marginally significant cluster was located in the left inferior parietal lobule (IPL, *x* = −48, *y* = −37, *z* = 31; see Supplementary Table 3 and Supplementary Figure). While this result suggests that IPL might be sensitive to motor training above and beyond audiovisual training, we are reluctant to draw strong conclusions from this result due to its failure to meet the FWE-corrected statistical threshold. As such, we do not discuss this result further in the main text, but consider some possibilities for its involvement in this task in the text accompanying the Supplementary Figure. The inverse contrast did not reveal any suprathreshold activations at the stringent cluster-corrected threshold or at a more liberal threshold of *P* < 0.001 and 10 voxels.

## Discussion

The main objective of the present study was to systematically investigate how layering action experience influences perception of whole-body movements. We predicted that, during action observation, the more modalities through which an observer has experienced a movement, the more sensorimotor brain regions should be engaged. Our findings broadly support this hypothesis. Specifically, we demonstrated progressively robust AON activity as action representations become increasingly rich, building up from no experience in the untrained condition to auditory experience only to visual and auditory experience and finally to motor, visual, and auditory experience. Within brain regions associated with the AON, this pattern of activity was most pronounced within PMC and the IPC. A similar pattern of increasingly robust performance scores the more modalities through which an action has been learnt emerged in the behavioral results, although the findings clearly demonstrate highest scores when dancing sequences from the PVA condition relative to the other three. In other words, PVA training was associated with the best performance, and both neural responses and behavioral performance showed a pattern of monotonic ascent as the number of training modalities increased from zero to three. Below we consider in more depth how these findings advance our understanding of the impact of action experience on perception.

### Multiple Routes to Action Learning

The data illustrated in Figure 4 suggest that core AON regions, as well as portions of the cingulate cortex, reflect the richness of a learner's experience with a previously novel action sequence. Moreover, this pattern of findings supports the notion that AON activity acts, in part, as an index of embodiment, with observation of physically, visually, and auditorily experienced movements resulting in the strongest AON activity out of the training modalities examined in this study (cf., Calvo-Merino et al. 2005; Cross et al. 2006; Aglioti et al. 2008).

These results underscore the malleability of neural responses with even minimal training manipulations, and also add support to theories that favor experience-dependent plasticity of the human brain (Dayan and Cohen 2011). In the present study, we provide evidence that after 1 week of training with complex, whole-body motor sequences, neural responses during action observation are impacted in an experience-dependent manner. Returning to the flamenco example in the introduction, we were particularly curious as to whether evidence would emerge to suggest that experience listening to the same kind of music (in the auditory-only condition) that accompanies full-body movement sequences (in the visual and auditory and the physical, visual, and auditory conditions) would also impact performance or AON engagement. As the results illustrated in Figures 3 and 4 attest, we find little evidence to support this. However, the present study remains the first to examine whether limited experience with the melody and rhythm that accompany complex action sequences results in learning that is reflected in AON engagement, despite the fact no specific action is implied by the music.

Ample opportunities remain for future work to further examine the relationship between sound and movement when the 2 are not causally linked. For example, it would be telling if pairing an auditory condition (random sound tracks) with visual or kinesthetic training (action observation or action performance) leads to increased AON activity after paired training. One could claim that the auditory condition in such an experiment would provide a baseline measurement that confirms that auditory training of nonaction-related sounds does not evoke AON activity. Regardless of the lack of strong support for auditory experience shaping AON engagement found in the current study, if we return to Hebbian learning theory, the present findings support the hypothesis that increasing the number of modalities through, which an action has been learned should increase the number of Hebbian associations (Hebb 1949; Keysers and Gazzola 2014). As Hebbian learning theory would predict, we find some evidence that increasing the number of sensory modalities through, which an action is learned leads to increasingly broad engagement of sensorimotor cortices during action perception, which in turn is associated with better physical performance of an action.

### Comparing the Impact of PVA and VA Experience

While prior studies have compared physical and observational action experience that individuals have acquired in their daily lives or on the basis of their profession (Calvo-Merino et al. 2006; Liew et al. 2013), fewer have implemented training manipulations that precisely quantify the amount of exposure to both kinds of experience and performance ability. Moreover, the degree to which any brain area represents both kinds of experience is often overlooked in such studies. In the present study, the PVA and VA training conditions exposed participants to an identical amount of visual and auditory experience with novel action sequences. A conjunction analysis of these 2 conditions revealed 4 regions common to physical and observational experience: PMC, IPC, SFG, and posterior cingulate cortex.

Overlap within premotor and parietal cortices for physical and observational experience was also reported by Cross et al. (2009), who interpreted this common pattern of activity as support for Blandin and Proteau's (2000) suggestion that observational practice works by engaging the same brain regions as physical practice. The present results further corroborate this interpretation. Left SFG engagement has been demonstrated during the selection and combination of motor representations during action observation (Buccino et al. 2004; Vogt et al. 2007). Even though participants were not performing a task-relevant motor task in the present study, it is possible that the SFG activity observed when watching PVA and VA sequences also relates to the accessing newly learned motor representations. The influence of expertise on posterior cingulate activation is consistent with the involvement of the cingulate cortex in episodic memory (Burgess et al. 2001; Leech and Sharp 2014). One possibility is that the posterior cingulate activity observed in the present study reflects engagement of such memory processes when participants observe sequences associated with either PVA or VA experience. This interpretation is consistent with what Calvo-Merino et al. (2005) and Cross et al. (2006) reported in their studies on action expertise.

Returning to the role of PMC, correlations between performance and activity within this region suggest another way in which premotor activity might serve as an index of embodiment. When participants' post-training dance scores from the PVA and VA conditions were correlated with BOLD responses within the 4 brain regions common to PVA and VA experience, the strength of signal within PMC only significantly correlated with how well participants performed sequences from the PVA or VA conditions in the post-training dance test only (notice, however, that the magnitude of the premotor response was not correlated to participants' initial score on the PVA sequences on training day 1; see blue diamonds in Fig. 6). This suggests that the positive correlations between premotor activity and performance scores that emerge after training are experience-specific and not (only) related to participants' aptitude at playing the dance video game. (We also correlated the amplitude of BOLD signal with training gains [post-test score from PVA or VA condition minus day 1 dance training score from PVA condition] and did not find any hint of a relationship between learning gains and amplitude of neural response [all *P* > 0.1].) Thus, it appears that the relationship might be due to some kind of overall competency at performing after training, and not the amount of learning that has taken place, per se). The fact that this part of PMC showed a greater response the better participants performed physically or observationally trained dance sequences is a novel finding that complements and extends prior reports of PMC engagement correlated with performance on physically trained tasks (Cross et al. 2009) and the degree of expertise with an action when listening to action-related sentences (Beilock et al. 2008).

Concerning correlations within the other 3 brain regions that emerged from the conjunction analysis, we might have expected the response amplitude of IPS to also positively correlate with participants' performance aptitude, based on Frey and Gerry's (2006) finding that activity within the right intraparietal sulcus predicts accuracy with which observed actions are subsequently performed. In the present study, left IPC showed a trend of this relationship for PVA sequences, but the correlation failed to reach significance (*P* = 0.065). Taken together, our results suggest that left PMC is most sensitive to how well an action is learned or embodied, whether via physical and observational experience, and that participants' relative performance can be predicted based on the strength of signal within this region during action observation.

### Possible Functional Relevance of Increased AON Sensitivity to Multisensory Trained Actions

Although the present study was not designed to directly assess the functional relevance of increased AON sensitivity to multisensory trained actions, some consideration of the broader theoretical significance of this pattern of findings should nonetheless inform understanding of the interplay between action and perception in the human brain. Recent theoretical papers propose that the shaping of sensorimotor brain regions by experience is related to the refining of active inference processes, by which an observer is better able to anticipate or predict the outcome of an observed other's actions (Keysers and Gazzola 2014; Press et al. 2011). To date, this work has focused less on the extent to which unimodal or unisensory experience might influence such active inference processes compared with multisensory experience. When the current result are considered in light of this literature, it seems plausible that increased sensorimotor engagement when viewing actions with which observers have greater sensorimotor experience relates to increasingly refined prediction or inference about how a trained action will unfold, relative to an untrained action (see also Calvo-Merino et al. 2006). Future studies specifically designed to address the functional relevance of AON sensitivity to unimodal versus multimodal training experience will help to determine with greater precision the extent to which increased sensorimotor engagement assists us in understanding others movements.

### Limitations and Future Directions

One feature of the present study worth noting is that animated avatars, not real humans, performed all movements. Studies comparing perception of actions performed by real humans compared with avatars (Thompson et al. 2011) or animated robots (Shimada 2010; Cross, Liepelt et al. 2012; Urgen et al. 2013) report mixed findings regarding sensorimotor engagement when watching actions performed by artificial humanoid agents compared with real humans. For example, Shimada (2010) found attenuated AON engagement when watching actions performed by nonhuman agents, while Cross, Liepelt et al. (2012) reported greater AON engagement when watching robotic compared with human actions. While we acknowledge that, using humanoid avatars instead of real humans in the present study could have influenced our results (although conflicting prior evidence makes it difficult to speculate how, precisely), this possibility should not compromise our findings for 2 reasons. First, as all conditions (UNT, A, VA, and PVA) featured actions performed by avatars, the relative differences between the training conditions remain informative for how different kinds of experience impact action perception. Second, prior work demonstrates that visual or motor experience with alternative displays of human movement (such as actions performed by point light walkers) impacts visual sensitivity to these movements (Jacobs et al. 2004). Participants in the present study acquired a considerable amount of visual and motor experience with the “Dance Central” avatars by the post-training fMRI session and final dance test. Consequently, watching actions performed by these particular avatars should have been generally familiar after several days of training and, therefore, AON engagement is more likely to reflect differences in training experience rather than a response to the novelty of seeing actions performed by animated avatars.

Another limitation concerns the impact that providing feedback in the PVA training condition only might have had on learning, performance, and neural engagement during the post-training scan. Behavioral evidence demonstrates enhanced learning on a range of tasks when participants receive timely feedback about their performance (Salmoni et al. 1984). Participants could see their overall dance performance score after each dance sequence, so it is likely they could see that they were improving day after day (if they remembered their scores). In contrast, in the VA and A conditions, they were not given any feedback about their performance. While we did not specifically draw participants' attention to their scores in the PVA condition, we would urge future studies in this area to more closely control the impact of feedback by either eliminating all access to feedback or providing commensurate amounts and quality of feedback across all training conditions.

A final limitation to consider, which also provides rich grounds for future work, concerns the restricted number and combination of sensory modalities investigated. One factor constraining the range of possible experience conditions we could study was the technical limitations of the video game setup used for training and performance monitoring (i.e., it would not be possible to investigate physical experience only with the present setup). Working within these limitations, we specifically chose experience categories that ranged from as minimal and action-unspecific as possible to as multimodal and action-specific as possible, and combined them to create training conditions with increasing amounts of sensory information with novel, whole-body movement sequences. Naturally, exploring other unimodal conditions and multimodal combinations not investigated in the present study would be valuable to determine whether action representations acquired via visual experience only, physical experience only, physical and auditory experience, or via physical and visual experience also engage left PMC and other AON regions according to the pattern of findings reported here.

**Figure 7.**
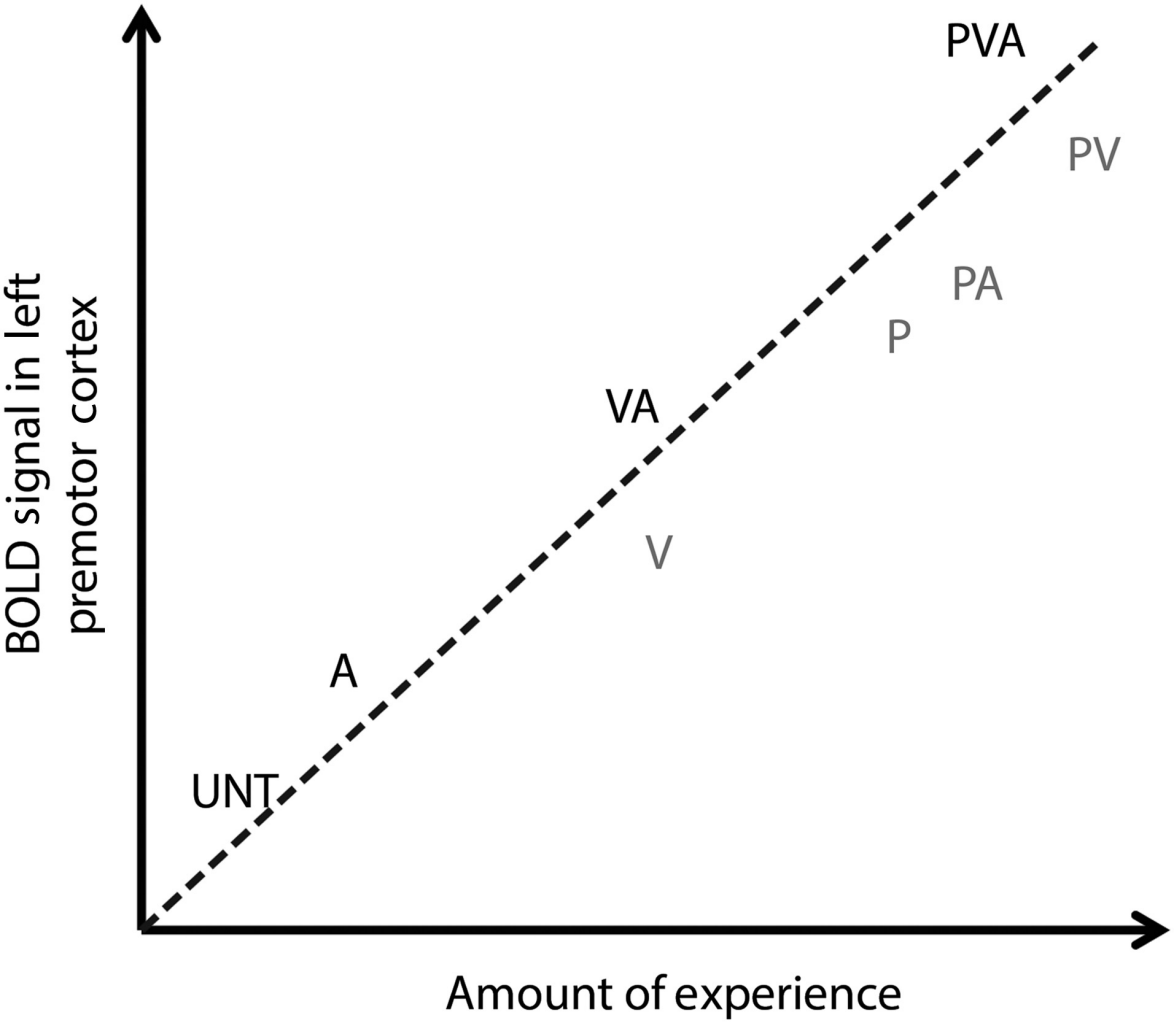
A hypothesized relationship between BOLD response in the left premotor area and the amount of experience and number of modality experienced. UNT, untrained; A, audio; V, visual; VA, visual + audio; P, physical; PA, physical + audio; PV, physical + visual; PVA, physical + visual + audio experience. In boldface font, the BOLD response from UNT, A, VA, and PVA training manipulations are deduced from the present study, whereas in gray V, P, PA, and PV experience level and associated BOLD signal are hypothesized and require further exploration.

Based on the amount and quality of information available about an action from the auditory, visual, and motor modalities, we would expect these new conditions to fit into our current findings in an increasing pattern of premotor signal (and behavioral performance) along the lines of UNT < A < V < VA ≤ P < PA < PV < PVA (see Fig. 7 for an illustration of this hypothetical model). A rich literature documenting the impact of visual-only experience on AON engagement (e.g., Mattar and Gribble 2005; Frey and Gerry 2006; Liew et al. 2013) supports the prediction that action information provided by unimodal visual experience should exceed that of unimodal auditory experience, thus resulting in greater AON engagement. Along the same lines, we would expect physical experience only to engage the AON at an intermediate level between audiovisual and visuomotor experience (cf., Casile and Giese 2006). It is worth noting that while unimodal visual experience is often used to study how experience shapes AON engagement (Calvo-Merino et al. 2006; Cross et al. 2009; Liew et al. 2013), a comparison between visual and audiovisual experience may provide more ecologically valid evidence of a Hebbian learning account of the impact of layering experience (since visual-only conditions are typically used for AON studies, rather than auditory-only, as in the current study). More generally speaking, if the hypothetical model proposed in Figure 7 were validated by future studies examining the conditions not included in the present experiment, this would add further support to a Hebbian learning view of AON engagement, where more sensory information about an action should lead to more Hebbian associations and thus increased activity of the implicated regions (Keysers and Gazzola 2014). However, these ideas remain speculative at this stage, as the present study tested only 4 of 8 possible combinations of conditions. A challenge for future work will thus be to determine whether PMC does indeed act as a compiler of sensory information during novel action learning, such that increasing exposure to action information results in increasing levels of activity.

## Summary and Conclusions

We have shown how novel action learning acquired via auditory, visual, and motor domains influences brain activity during action observation, as well as behavioral performance, depending on the number of modalities through which a new action was learned. This is the first study to systematically investigate how adding layers of sensory experience shapes sensorimotor cortical responses when watching complex whole-body movements. Both physical and observational experience were found to shape responses within core AON regions in a similar way. Moreover, a portion of the left PMC was particularly sensitive to the layering of experience, as the strength of signal within this region positively correlated with participants' ability to physically reproduce an observed action. Taken together, these data illuminate the extent to which different kinds of experience with an action, as well as one's own physical abilities, shape the way we perceive others in action.

## Supplementary Material

Supplementary material can be found at: http://www.cercor.oxfordjournals.org/.

## Funding

This work was supported by the Economic and Social Research Council (ES/K0001892/1); the Netherlands Organisation for Scientific Research (Veni Award 451-11-002); and a Marie Curie Career Integration Grant (PCIG11-GA-2012-322256) to E.S.C. Funding to pay the Open Access publication charges for this article was provided by an ESRC grant to E.S.C. (ES/K001892/1) and from Bangor University's RCUK Open Access Block Grant.

## Supplementary Material

Supplementary Data
