# Supplementary material for: Additive Routes to Action Learning: Layering Experience Shapes Engagement of the Action Observation Network
Source: Cereb Cortex. 2015 Jul 24;25(12):4799–811. doi: 10.1093/cercor/bhv167 (PMC4635920; doi:10.1093/cercor/bhv167)
Supplement: Supplementary Data [file supp_25_12_4799__index.html]

Additive Routes to Action Learning: Layering Experience Shapes Engagement of the Action Observation Network — Additive Routes to Action Learning: Layering Experience Shapes Engagement of the Action Observation Network — Supplementary Data 

# Additive Routes to Action Learning: Layering Experience Shapes Engagement of the Action Observation Network

## Supplementary Data

Supplementary Data

- Supplementary Figure - docx file
- Supplementary Tables - docx file
