# Supplementary material for: Additive Routes to Action Learning: Layering Experience Shapes Engagement of the Action Observation Network
Source: Cereb Cortex. 2015 Jul 24;25(12):4799–811. doi: 10.1093/cercor/bhv167 (PMC4635920; doi:10.1093/cercor/bhv167)
Supplement: Supplementary Data [file supp_bhv167_bhv167supp_fig.docx]

**Supplementary Figure.** Activation in the left inferior parietal lobule (IPL) associated with the observation of PVA trained sequences compared with VA trained sequences. Plots represent the parameter estimates in IPL for both PVA and VA conditions and are included for illustration purposes only. Error bars indicate the across-subject standard error of the mean.


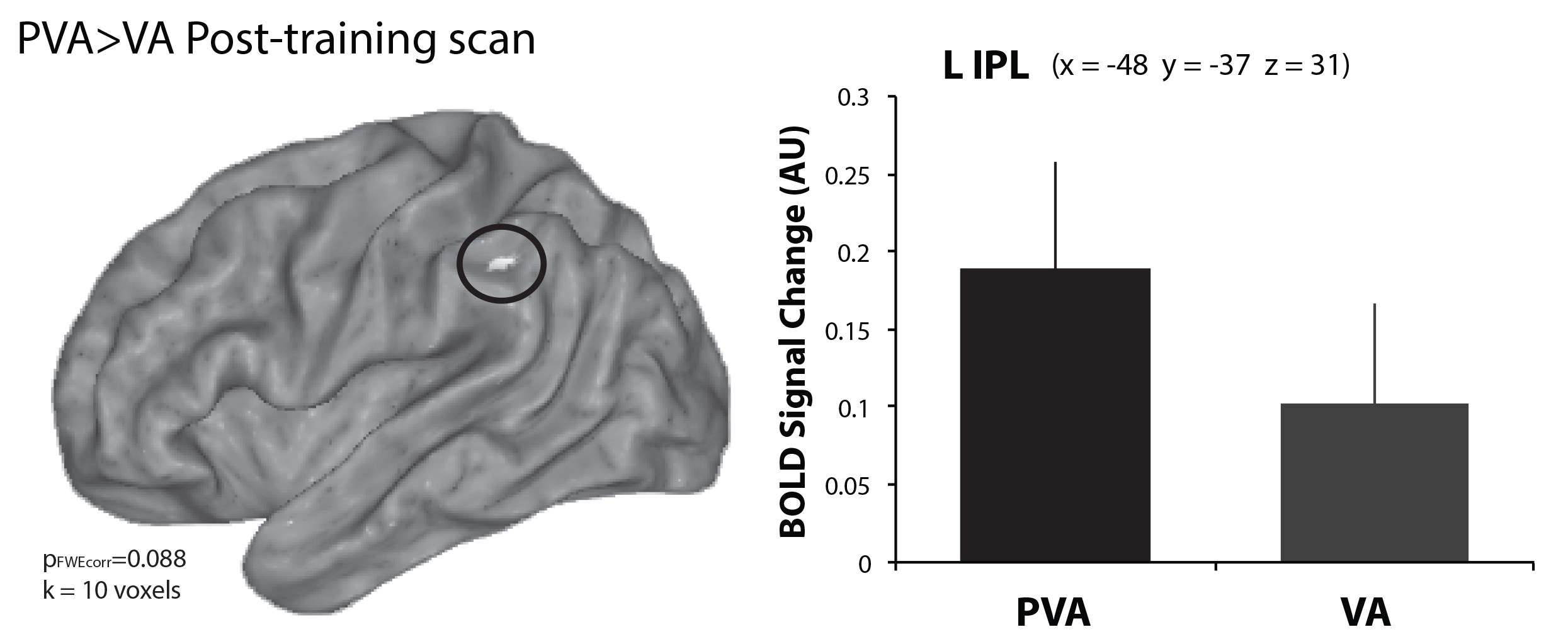


*What is special about physical experience?*

The current design enabled us to investigate what is unique to *physically* practicing an action by directly comparing the PVA and VA training conditions. This contrast allows us to effectively subtract the impact of the visual and auditory cues and ask what is special about motor experience with a new action**. While no brain regions met the cluster-corrected threshold for this contrast, a brain region within the left inferior parietal lobule emerged from this contrast at a marginally significant value of *p_corr_* = 0.088. While the lack of power means this result must be interpreted with caution, prior literature nonetheless informs what this region might be coding in the current study.** Tranel and colleagues (2003) showed the importance of IPL in action perception by testing patients with an IPL lesion, whose action recognition was impaired (for a recent meta-analysis on this topic, see Urgesi et al., 2014). In an earlier study, Grèzes and colleagues (1999) found that activity in IPL was greater when participants observed meaningless actions learned via visuomotor practice compared to unlearned, meaningless actions. Subsequently, Calvo-Merino and colleagues reported greater IPL activity when participants observed movements from their own motor repertoire, compared to kinematically similar but unfamiliar movements (Calvo-Merino et al., 2005). A related study also demonstrated that IPL activity during action observation is parametrically modulated by participants’ ability to physical perform observed actions (Cross et al., 2006). The current study is the first to show that activity in IPL is modulated by the *type* of experience, *per se*, in that the IPL response was particularly sensitive to physical experience. **This finding reinforces the idea that IPL integrates motor with audiovisual experience and that newly-acquired action representations are evoked during observation in the absence of overt movement (c.f. Cross et al., 2012). Such a finding also ties in well with a rich literature linking IPL to action affordances, how to perform an action, and action kinematics (e.g., Fagg & Arbib, 1998; Schubotz et al., 2014). This literature suggests the IPL codes the practical knowledge of how to move, which in the current study is likely greater when participants have physically practiced a movement sequence. Again, however, given the marginally significant nature of the statistics in the current study, it remains to be seen whether this result is replicated reliably in future investigations.**

REFERENCES

Calvo-Merino B, Glaser DE, Grèzes J, Passingham RE, Haggard P. 2005. Action observation and acquired motor skills: an FMRI study with expert dancers. Cereb. Cortex. 15:1243–1249.

Cross ES, Hamilton AF, Grafton ST. 2006. Building a motor simulation de novo: observation of dance by dancers. Neuroimage. 31:1257–1267.

Cross ES, Cohen NR, Hamilton AF, Ramsey R, Wolford G, Grafton ST. 2012. Physical experience leads to enhanced object perception in parietal cortex: insights from knot tying. Neuropsychologia. 50(14):3207-17.

Grèzes J, Costes N, Decety J. 1999. The effects of learning and intention on the neural network involved in the perception of meaningless actions. Brain. 122(10):1875-87.

Fagg AH and Arbib MA. 1998. Modeling parietal-premotor interactions in primate control of grasping. Neural Netw. 11(7-8):1277-1303.

Schubotz RI, Wurm MF, Wittmann MK, von Cramon DY. Objects tell us what action we can expect: dissociating brain areas for retrieval and exploitation of action knowledge during action observation in fMRI.

Tranel D, Kemmerer D, Adolphs R, Damasio H, Damasio AR. 2003. Neural correlates of conceptual knowledge for actions. Cogn Neuropsychol. 20(3):409-32.

Urgesi C, Candidi M, Avenanti A. 2014. Neuroanatomical substrates of action perception and understanding: an anatomic likelihood estimation meta-analysis of lesion-symptom mapping studies in brain injured patients. Front. Hum. Neurosci. 8:344.
