# Supplementary material for: Additive Routes to Action Learning: Layering Experience Shapes Engagement of the Action Observation Network
Source: Cereb Cortex. 2015 Jul 24;25(12):4799–811. doi: 10.1093/cercor/bhv167 (PMC4635920; doi:10.1093/cercor/bhv167)
Supplement: Supplementary Data [file supp_bhv167_bhv167supp_tables.docx]

**SUPPLEMENTARY TABLES**

**Supplementary Table 1**. Regions associated with an increase of experience and modalities involve in the training, across scan session (Day 5 > Day 1).

| Region | BA | MNI Coordinates | | | Putative Functional Name | *t*-value | Cluster Size | P_corr._ Value |
| --- | --- | --- | --- | --- | --- | --- | --- | --- |
|  |  | x | y | z |  |  |  |  |
| **L anterior cingulate** | **24/32** | **-15** | **32** | **28** |  | **7.01** | **296** | **<0.001** |
| **R superior frontal gyrus** | **8/9** | **21** | **29** | **31** | **SFG** | **5.66** |  |  |
| **R superior frontal gyrus** | **9** | **15** | **41** | **28** | **SFG** | **5.34** |  |  |
| **L cingulate cortex/calcarine gyrus/** | **18** | **-12** | **-58** | **10** |  | **6.85** | **931** | **<0.001** |
| **R lingual gyrus** | **17/18** | **9** | **-55** | **7** |  | **6.84** |  |  |
| **Precuneus** | **31** | **0** | **-67** | **28** | **SPL** | **5.93** |  |  |
| **L middle occipital gyrus** | **39** | **-45** | **-73** | **25** | **IPC** | **6.22** | **413** | **<0.001** |
| **L inferior parietal lobule** | **7** | **-39** | **-55** | **46** | **IPL** | **4.42** |  |  |
| **L angular gyrus** | **39** | **-57** | **-58** | **25** | **IPC** | **4.33** |  |  |
| **R middle occipital gyrus** | **39** | **36** | **-67** | **37** | **SPL** | **6.07** | **130** | **0.005** |
| **R middle occipital gyrus** | **19** | **39** | **-70** | **28** | **IPC** | **4.82** |  |  |
| **R middle temporal gyrus** | **39** | **54** | **-64** | **22** | **MTG** | **4.72** |  |  |
| **L fusiform gyrus** | **20** | **-24** | **-34** | **-20** | **PHG** | **6.00** | **74** | **0.042** |
| **L fusiform gyrus** | **20** | **-30** | **-25** | **-26** | **PHG** | **4.67** |  |  |
| **L fusiform gyrus** | **20** | **-36** | **-43** | **-26** |  | **3.60** |  |  |
| **L inferior frontal gyrus** | **44** | **-48** | **14** | **34** | **PMC** | **5.82** | **649** | **<0.001** |
| **L inferior frontal gyrus** | **45** | **-51** | **23** | **16** | **IFG** | **5.60** |  |  |
| **L inferior frontal gyrus** | **44** | **-51** | **8** | **22** | **IFG** | **5.56** |  |  |
| **L temporal pole** | **38** | **-45** | **14** | **-20** | **STG** | **5.71** | **110** | **0.010** |
| **L inferior frontal gyrus** | **38** | **-27** | **11** | **-23** |  | **4.75** |  |  |
| **L medial temporal pole** | **38** | **-39** | **14** | **-29** |  | **4.23** |  |  |
| R middle frontal gyrus | 6 | 36 | 11 | 43 | DLPFC | 5.07 | 46 | 0.150 |
| L pons | 36 | -9 | -25 | -26 |  | 5.04 | 29 | 0.416 |
| Pons | 36 | 0 | -37 | -35 |  | 4.21 |  |  |
| **R posterior cingulate** | **23** | **-9** | **-34** | **28** |  | **4.88** | **188** | **<0.001** |
| **R cingulate gyrus** | **23** | **6** | **-16** | **28** |  | **4.65** |  |  |
| **R posterior cingulate** | **23** | **6** | **-28** | **28** |  | **4.46** |  |  |
| R temporal pole | 38 | 39 | 11 | -29 | STG | 4.68 | 32 | 0.295 |
| L dorsolateral prefrontal | 8 | -6 | 14 | 46 | SMA | 4.55 | 35 | 0.255 |
| L mid orbital gyrus | 10 | -12 | 59 | -2 | SFG | 4.47 | 47 | 0.143 |
| R mid orbital gyrus | 10 | 9 | 62 | -5 |  | 4.23 |  |  |
| Anterior cingulate | 24 | 0 | 17 | 22 |  | 4.32 | 49 | 0.130 |
| Anterior cingulate | 24 | 0 | 8 | 28 |  | 3.99 |  |  |
| Anterior cingulate | 24 | 9 | 17 | 22 |  | 3.90 |  |  |
| L middle temporal gyrus | 22 | -54 | -10 | -20 |  | 4.25 | 39 | 0.210 |
| R middle frontal gyrus | 44 | 45 | 29 | 22 | IFG | 4.18 | 50 | 0.124 |
| R inferior frontal gyrus | 44 | 33 | 14 | 19 | IFG | 4.14 |  |  |
| R inferior frontal gyrus | 44 | 42 | 14 | 28 | IFG | 3.90 |  |  |
| R fusiform gyrus | 37 | 27 | -43 | -17 |  | 3.92 | 10 | 0.795 |

BA, Brodmann’s area; R: right, L: left; DLPFC: dorsolateral prefrontal cortex; IFG, inferior frontal gyrus; IPL, inferior parietal lobule; IPS: intraparietal sulcus; LO: lateral-occipital region; PHG, parahippocampal gyrus; MFG, middle frontal gyrus; MTG, middle temporal gyrus; SFG: Superior frontal gyrus; SFS: superior frontal sulcus; SPL: superior parietal sulcus; STG, superior temporal gyrus; STS, superior temporal sulcus. Significance at all sites for each contrast was tested by a one-sample t-test on beta values averaged over each voxel in the cluster, P < 0.001, uncorrected; k=10voxels. BOLD regions are FWE-cluster corrected. Up to three local maxima are listed when a cluster has multiple peaks more than 8 mm apart.

**Supplementary Table 2.** Regions associated with the interaction between training experience (physical, audiovisual, audio with untrained) and test session (pre-training scan; post-training scan).

| Region | BA | | MNI Coordinates | | | Putative Functional Name | *t*-value | Cluster Size | *p_corr_* value |
| --- | --- | --- | --- | --- | --- | --- | --- | --- | --- |
|  |  | | x | y | z |  |  |  |  |
| ***(a) PVA*** ***Training> Untrained ( Post Training) > PVA Training> Untrained ( Pre Training)*** | | | | | | | | | |
| **L inferior frontal gyrus** | | **45** | **-48** | **14** | **31** | **PMv** | **6.68** | **652** | **<0.001** |
| **L inferior frontal gyrus** | | **44/6** | **-48** | **23** | **16** |  | **6.64** |  |  |
| **L inferior frontal gyrus** | | **44** | **-51** | **8** | **22** |  | **5.79** |  |  |
| **R precuneus** | | **17** | **9** | **-52** | **7** | **rACC** | **6.58** | **861** | **<0.001** |
| **L calcarine gyrus** | | **18** | **-12** | **-58** | **10** |  | **6.04** |  |  |
| **L precuneus** | | **31** | **0** | **-67** | **28** |  | **5.79** |  |  |
| **L middle occipital gyrus** | | **39** | **-42** | **-73** | **25** | **IPC** | **5.85** | **159** | **0.001** |
| **L temporal pole** | | **38** | **-45** | **14** | **-20** | **STG** | **5.72** | **82** | **0.027** |
| **L inferior frontal gyrus** | | **38** | **-27** | **11** | **-23** |  | **4.48** |  |  |
| **L medial temporal pole** | | **38** | **-39** | **11** | **-29** |  | **3.94** |  |  |
| **R superior frontal gyrus** | | **32** | **15** | **41** | **28** | **SFG** | **5.67** | **159** | **0.001** |
| **R middle frontal gyrus** | | **9** | **24** | **50** | **28** |  | **4.71** |  |  |
| **R superior frontal gyrus** | | **32** | **21** | **29** | **31** |  | **4.61** |  |  |
| **R middle occipital gyrus** | | **19** | **36** | **-67** | **37** | **SPL** | **5.40** | **86** | **0.023** |
| **R middle occipital gyrus** | | **19** | **39** | **-70** | **28** | **IPC** | **4.09** |  |  |
| L posterior precentral gyrus | | 4 | -24 | -19 | 46 | PMd | 5.36 | 19 | 0.544 |
| L anterior cingulate | | 32 | -15 | 32 | 28 | rACC | 5.32 | 35 | 0.247 |
| L middle frontal gyrus | | 32 | -21 | 44 | 28 |  | 3.83 |  |  |
| **L middle frontal gyrus** | | **6** | **-27** | **5** | **46** | **PMd** | **5.27** | **113** | **0.008** |
| R middle frontal gyrus | | 6 | 39 | 11 | 43 | Premotor | 4.98 | 42 | 0.174 |
| **R inferior frontal gyrus** | | **45** | **45** | **26** | **22** | **MFG** | **4.68** | **69** | **0.048** |
| **R inferior frontal gyrus** | | **45** | **42** | **14** | **28** |  | **4.02** |  |  |
| **L posterior cingulate gyrus** | | **23** | **-6** | **-31** | **25** |  | **4.67** | **239** | **<0.001** |
| **R posterior cingulate** | | **23** | **6** | **-16** | **28** |  | **4.62** |  |  |
| **R cingulate** | | **24** | **6** | **-7** | **31** |  | **4.59** |  |  |
| R temporal pole | | 38 | 45 | 14 | -20 | STG | 4.54 | 25 | 0.407 |
| R temporal pole | | 38 | 36 | 11 | -29 |  | 4.40 |  |  |
| L superior frontal gyrus | | 8 | -3 | 14 | 46 | SMA | 4.41 | 25 | 0.407 |
| L middle temporal gyrus | | 22 | -51 | -10 | -20 | MTG | 4.34 | 21 | 0.368 |
| L inferior parietal lobule | | 7 | -39 | -55 | 46 | IPL | 4.30 | 66 | 0.055 |
| L inferior parietal lobule | | 7 | -45 | -46 | 52 | IPL | 4.00 |  |  |
| L middle temporal gyrus | | 40 | -57 | -55 | 22 | IPC | 4.13 | 22 | 0.471 |
| ***(b) VA Training> Untrained (Post Training)*** *>* ***VA Training> Untrained*** *(****Pre Training)*** | | | | | | | |  |  |
| **L calcarine gyrus** | **30** | | **-9** | **-61** | **16** |  | **6.73** | **344** | **<0.001** |
| **L calcarine gyrus** | **18** | | **-9** | **-70** | **19** |  | **5.93** |  |  |
| **R calcarine gyrus** | **30** | | **9** | **-61** | **16** |  | **5.27** |  |  |
| **L middle frontal gyrus** | **9** | | **-21** | **32** | **37** |  | **6.07** | **190** | **0.001** |
| **L superior frontal gyrus** | **8** | | **-15** | **17** | **46** | **pre-SMA** | **4.77** |  |  |
| **L superior frontal gyrus** | **8** | | **-15** | **26** | **52** | **pre-SMA** | **4.16** |  |  |
| **L posterior cingulate** | **19** | | **-12** | **-40** | **28** |  | **5.14** | **35** | **0.278** |
| L superior frontal gyrus | 10 | | -15 | 56 | 13 |  | 4.98 | 22 | 0.495 |
| L pons – cerebellum | 36 | | -12 | -28 | -29 |  | 4.80 | 32 | 0.318 |
| Pons | 36 | | 0 | -37 | -35 |  | 4.38 |  |  |
| L fusiform gyrus | 20 | | -30 | -40 | -17 |  | 4.75 | 17 | 0.610 |
| **L middle occipital gyrus** | **39** | | **-39** | **-70** | **25** | **IPC** | **4.73** | **91** | **0.027** |
| **L middle temporal gyrus** | **39** | | **-54** | **-64** | **22** |  | **4.17** |  |  |
| **L middle occipital gyrus** | **19** | | **-33** | **-67** | **31** | **IPC** | **4.05** |  |  |
| R superior frontal gyrus | 6/8 | | 27 | 17 | 43 | SFG | 4.48 | 21 | 0.516 |
| R superior frontal gyrus | 10 | | 21 | 50 | 31 |  | 4.38 | 11 | 0.766 |
| R anterior cingulate gyrus | 32 | | 18 | 29 | 28 |  | 4.35 | 15 | 0.661 |
| R superior medial gyrus | 9 | | 3 | 59 | 25 | SFG | 4.09 | 16 | 0.635 |
| L superior mdeial gyrus | 9 | | -6 | 56 | 28 | SFG | 3.95 |  |  |
| R middle occipital gyrus | 19 | | 36 | -67 | 37 | SPL | 4.05 | 10 | 0.792 |
| ***(c) A Training > Untrained ( Post Training) > A Training > Untrained (Pre Training)*** | | | | | | | | |  |
| L inferior frontal gyrus |  | | -30 | 35 | 10 |  | 5.77 | 11 | 0.766 |
| L caudate |  | | -27 | -19 | 25 |  | 5.15 | 31 | 0.286 |
| L cingulate gyrus |  | | -21 | -25 | 31 |  | 4.30 |  |  |
| L precentral gyrus | 3 | | -30 | -22 | 46 | M1 | 4.86 | 30 | 0.301 |
| R inferior frontal gyrus | 46 | | 39 | 35 | 7 |  | 4.18 | 15 | 0.645 |
| R cuneus | 18 | | 12 | -67 | 25 |  | 3.98 | 19 | 0.533 |

Results from the interaction analyses that assessed regions responsive to training experience and scan session. In bold are the regions FWE cluster corrected. BA, Brodmann’s area; R: right, L: left; IPL, inferior parietal lobule; IPC: intraparietal cortex; M1, primary motor cortex; MTG, middle temporal gyrus; PMv, ventral premotor area; PMd: dorsal premotor area; pre-SMA: pre-supplementary motor area; SMA: supplementary motor area; SFG, Superior frontal gyrus; SPL: superior parietal lobule; STG, superior temporal gyrus; rACC: rostral anterior cingulate cortex. Significance at all sites for each contrast was tested by a one-sample t-test on beta values averaged over each voxel in the cluster, P < 0.001, uncorrected; k=10voxels. BOLD regions are FWE-cluster corrected. Up to three local maxima are listed when a cluster has multiple peaks more than 8 mm apart.

**Supplementary Table 3.** Regions associated with the main effects between training experiences (PVA and VA) on day 5 (post training session).

| Region | BA | MNI Coordinates | | | Putative Functional Name | *t*-value | Cluster Size | *p_corr._* Value |
| --- | --- | --- | --- | --- | --- | --- | --- | --- |
|  |  | x | y | z |  |  |  |  |
| ***PVA Training> VA Training*** *(Post Training scan)* | | | | | | | | |
| L inferior parietal lobule | 45 | -42 | -40 | 31 | IPL | 5.87 | 68 | 0.088 |
| L inferior frontal gyrus | 6 | -42 | 11 | 28 | IFG | 4.29 | 17 | 0.632 |
| R angular gyrus | 7 | 39 | -58 | 37 | IPS | 4.02 | 12 | 0.750 |
|  |  |  |  |  |  |  |  |  |
| ***VA Training> PVA Training*** *(Post Training scan)* | | | | | | | | |
| *No suprathreshold clusters emerged from this contrast* | | | | | | | | |

IPL: intraparietal lobule; IFG: inferior frontal gyrus; IPS: intraparietal sulcus.
